# Knowledge of Hepatitis B Virus Infection, Immunization with Hepatitis B Vaccine, Risk Perception, and Challenges to Control Hepatitis among Hospital Workers in a Nigerian Tertiary Hospital

**DOI:** 10.1155/2015/439867

**Published:** 2015-01-22

**Authors:** Olusegun Adekanle, Dennis A. Ndububa, Samuel Anu Olowookere, Oluwasegun Ijarotimi, Kayode Thaddeus Ijadunola

**Affiliations:** ^1^Department of Medicine, Obafemi Awolowo University/Obafemi Awolowo University Teaching Hospitals Complex, Ile-Ife 220005, Osun State, Nigeria; ^2^Department of Community Health, Obafemi Awolowo University/Obafemi Awolowo University Teaching Hospitals Complex, Ile-Ife 220005, Osun State, Nigeria

## Abstract

*Background*. Studies had reported high rate of hepatitis B infection among hospital workers with low participation in vaccination programmes, especially those whose work exposes them to the risk of HBV infection. The study assessed knowledge of hepatitis B virus infection, risk perception, vaccination history, and challenges to control hepatitis among health workers. *Methods*. A descriptive cross-sectional study. Consenting health care workers completed a self-administered questionnaire that assessed respondents' general knowledge of HBV, vaccination history and HBsAg status, risk perception, and challenges to control hepatitis. Data was analysed using descriptive and inferential statistics. *Results*. Three hundred and eighty-two health care workers participated in the study. There were 182 males and 200 females. The respondents comprised 94 (25%) medical doctors, 168 (44%) nurses, 68 (18%) medical laboratory technologists, and 52 (14%) pharmacists. Over 33% had poor knowledge with 35% not immunized against HBV. Predictors of good knowledge include age less than 35 years, male sex, being a medical doctor, previous HBsAg test, and complete HBV immunisation. Identified challenges to control hepatitis include lack of hospital policy (91.6%), poor orientation of newly employed health workers (75.9%), and low risk perception (74.6%). *Conclusion*. Hospital policy issues and low risk perception of HBV transmission have grave implications for the control of HBV infection.

## 1. Background

Hepatitis B virus (HBV) is a hepadnavirus. Chronic hepatitis B infection is endemic in Asia and Africa with more than 75% of the world's chronic HBsAg carriers being of Asian and African origins [1]. The burden of the virus is however a global one, even as black children from HBV endemic areas adopted by whites have been implicated in infection of white families [2]. In addition, HBV is transmitted by the sexual route [3] and marriage across races could expose people from low incidence areas to HBV. There is high prevalence of HBV infection among blacks [4], as well as high rate of infection among hospital workers [5]. Moreover, hospital workers have low participation in vaccination programmes, especially those whose work exposes them to the risk of HBV infection [6, 7].

A good knowledge of HBV virus means and modes of infection as well as adequate vaccination may reduce infection rate. The knowledge of HBV is generally low among the populace in a study carried out among Turkish community in Netherland [8]. On the other hand, studies carried out among health care workers in Sudan and Morocco revealed that most of them had a good knowledge of blood as a medium of infection but lacked adequate vaccine coverage [9, 10]. HBV could be transmitted through many other routes, and inadequate knowledge of HBV among health workers may reflect their behavioural pattern to vaccination and safety measures.

Presently, Obafemi Awolowo University Teaching Hospitals Complex has no written policy on hepatitis control; hence, there is no compulsion for health workers to take standard precaution against this deadly virus. Apart from the annual world hepatitis day marked in the hospital, little awareness is created to guard against this virus. Previous studies in Nigeria have focused on medical students and theatre and laboratory workers and few of such studies with limited number of participants on health workers in other areas of the hospital service. Few or no studies have been conducted among all health professionals in any hospital to assess their knowledge base of HBV. It therefore becomes necessary to conduct a baseline assessment of health workers knowledge of HBV and their risk perception and relate the findings to their behavioural pattern toward HBV prevention and hence the need for this study.

## 2. Methods

This was a descriptive cross-sectional study conducted at the Obafemi Awolowo University Teaching Hospitals Complex, Ile-Ife, Nigeria. The hospital is a 576-bed tertiary health centre, 200 kilometres northeast of Lagos. The study was conducted in two of the principal hospitals (Ife State Hospital and Wesley Guild Hospital) which serve as referral centres for the neighbouring states of Oyo, Kwara, Ondo, Ekiti, and Kogi.

The study population included the clinical members of staff, namely, doctors, nurses, pharmacists, and medical laboratory technologists. The study was conducted between January and April 2013.

The required sample size of 382 was calculated using an appropriate statistical formula for estimating the minimum sample size in descriptive health studies [*n* = *Z* ^2^ *pq*/*d* ^2^] [11], where 53.8% of health care workers completed HBV vaccination [7]. The total number of categories of clinical staff (i.e., medical doctors, nurses, laboratory technologists, and pharmacists) at the selected hospitals was collected at the establishment department. The number allocated to each group of clinical staff was determined proportionately using the formula:  *n*/*N* × 382, where *n* is the number of occupational groups and *N* is the total number of clinical staffs.

Eligible persons after an informed consent completed a self-administered pretested questionnaire. The questionnaire assessed the respondents' general knowledge of hepatitis B virus, mode of transmission, risk perception, and challenges to control HBV among respondents. For those with positive HBsAg serostatus, the questionnaire inquired about action taken. The questionnaire also assessed compliance with treatment among those who were on treatment as well as vaccination history of those who were negative to HBsAg. The questionnaires were distributed consecutively to members of each occupational group during the break period after completion of a written consent form. The respondents were allowed to fill the questionnaire in their spare time at their convenience. Questionnaire information was anonymised.

The Ethics and Research Committee of the Obafemi Awolowo University Teaching Hospitals Complex approved the study with protocol numbers IRB/IEC/0004553 and NHREC/27/02/2009a.

The data collected was entered and kept in a password-protected computer.

The data obtained was analysed using SPSS version 16. Simple descriptive and inferential statistics were done. Test of significance was conducted using appropriate statistical methods. Multivariate analysis was performed using logistic regression to evaluate sociodemographic variables and other variables that are independently associated with HBV knowledge as well as HBV vaccine uptake. Adjusted odd ratio (AOR) and 95% CI were presented and used as measures of the strength of association. Significant level was put at *P* < 0.05.

## 3. Results

Out of 500 health workers approached none declined participation but 90 did not return their questionnaire. 28 questionnaires were excluded from analysis because of noncompleteness. A total of 382 questionnaires with completed data were analysed (response rate of 76%). The mean age of the study participants was 33.8 ± 8.9 years (age range 20–59 years). There were 182 males and 200 females, with M : F ratio of about 1 : 1. The respondents are comprised of 94 (25%) medical doctors, 168 (44%) nurses, 68 (18%) medical laboratory technologists, and 52 (14%) pharmacists. The medical doctors included 45 (12%) house physicians, 44 (12%) resident doctors, and 5 (1%) consultant physicians/surgeons. Two hundred and sixteen (57%) were married, and the rest 166 (43%) were single. Most, 379 (99%), had tertiary level of education (Table 1). Reported prevalence of HBsAg among the respondents was 6.7%.

Majority, 367 (96%), of the participants were aware of HBV, and this was not statistically significant among the professional groups (doctors 93/94 (99%) versus nurses 159/168 (95%) versus lab technologists 64/68 (94%) versus pharmacists 51/52 (98%); *P* > 0.05). The knowledge of the transmission of HBV was good for blood as a medium for all categories of professionals. However, knowledge of other body fluids as source of infection varies among respondents. For instance, doctors had a reasonable good knowledge of saliva as a medium of infection 55/94 (59%) while this knowledge was poor among other professionals. On the other hand, only doctors had a poor knowledge that vaginal/seminal fluid could be a source of infection 25/94 (27%). Also the knowledge of exchange of used needles among patients as well as infected mothers with HBV transmitting the virus to their unborn babies was good for all professionals.

On the knowledge of the population at risk, nurses and laboratory technologists were the least aware that men having sex with men (MSM) could transmit HBV, 111/168 (66%) and 44/68 (65%), respectively, compared with the highest response among doctors 91/94 (98%), *P* < 0.05. Also laboratory technologists reported the least awareness of commercial sex workers 49/68 (72%), multiple sex partners 47/68 (69%), patients with sickle cell anaemia who have had multiple blood transfusions 19/68 (28%), and health care workers 59/68 (87%) as population at risk of HBV infection. This compared with 93/94 (99%), 91/94 (97%), 70/94 (75%), and 94/94 (100%) respectively, among doctors, *P* < 0.05. Also, pharmacists were the least who were aware that long distance drivers were at risk of HBV infection 13/52 (25%) compared with the other professionals, *P* < 0.05 (Table 2). All the respondents had reasonably good knowledge of the chronic complications of HBV. However, medical doctors had better knowledge of these chronic complications than the other professionals, *P* < 0.05 (Table 2).

In reported HBsAg test, nurses were the least group that knew their present HBsAg status 82/118 (70%). All the respondents were aware of hepatitis B vaccine with compliance with vaccination regime being the lowest among nurses than the other groups (87/168, 52%). Only 248 (65%) of our respondents were fully immunised with HBV vaccine. However, all participants were willing to receive HBV vaccine if given the opportunity (Table 3).

In the multivariate logistic regression model the predictors of good knowledge of HBV were age <35 years, male sex, being a medical doctor, previous HBsAg test, and complete immunisation with HBV vaccine (Table 4).

Also, factors determining HBV vaccine uptake among participants included male sex and having had a previous HBsAg test (Table 5).

Identified challenges to control hepatitis included inappropriate hospital policy (91.6%), poor orientation of newly employed health workers (75.9%), and low risk perception (74.6%) (Table 6).

## 4. Discussion

The study assessed knowledge of hepatitis B virus, risk perception, vaccination, and challenges to control hepatitis among health workers in a Nigerian tertiary hospital.

The reported prevalence of HBsAg was low in this study when most seroprevalence studies have between 10 and 15% of the Nigerian population being positive to HBsAg [12]. The reason for the low figure may have been because this is a questionnaire administered study rather than a seroprevalence study. Many people who think they are negative may be infected without a serotest. Besides, hepatitis infection is usually higher among health workers than the general population [5].

The awareness level of 96% for HBV among the respondents was similar to that reported by Okwara et al. [13]. This may probably have been as a result of the educational programmes on hepatitis received from the place of work and the news media as well as patients and staff members with complications of chronic hepatitis B virus infection that present regularly to the hospital. Some respondents did not know about the chronic complications of HBV like liver cirrhosis and liver cancer. This shows the lack of in-depth knowledge about HBV among these health workers beyond ordinary awareness. This finding agrees with the reports by other authors in Nigeria [13, 14].

There was a good knowledge of blood as a medium of infection in this study which is similar to reports by both Bakry and Djeriri et al., among Sudanese and Moroccans health workers [9, 10]. The knowledge of the respondents on saliva and tear was poor compared with blood as a medium. Kabir et al. have similarly noted a poor knowledge of the routes of HBV infection among Iranian medical specialists [15]. There was a poor knowledge of the vaginal route of HBV infection among doctors in this study contrary to reports in the literature. Studies have shown that HBV can be transmitted through the sexual route [3, 15, 16], as well as among family members in the household [16]. It thus seems that the major areas health workers lacked adequate knowledge is in the obscured routes of transmitting HBV.

The prevalence of HBsAg is increased in individuals with multiple sexual partners, sickle cell anaemic patients, long distance truck drivers, and injection drug users as well as men who have sex with men (MSM) [17–21]. The knowledge of these among health workers will call for caution and strict adherence to universal precaution among health workers having direct contact with them. However, in this survey, the result showed a poor knowledge of this among some categories of health workers. Only the doctors had a good knowledge of this. This finding contrasted with that by Kabir et al. among medical specialists [15].

The proportion of the respondents that ever screened for HBV infection was particularly low among the nurses and pharmacists. The implication of this is that health workers who are infected with HBV may present with any of the chronic complications of HBV such as hepatocellular carcinoma or liver cirrhosis as is usually observed in the general population. Furthermore, the implication of a negative HBsAg test was not known to some respondents as very few sought vaccination.

There seems to be a high level of vaccine awareness and low vaccination coverage among health workers in Nigeria. Only 54% of health workers completed HBV vaccination in this hospital in a previous HBV vaccination exercise [7], while 65% of respondents reported complete HBV vaccine in this study. This is despite the fact that the hospital carries out occasional vaccination programmes. This pattern is similar to reports from other centres: Kesieme in south-south geopolitical zone of Nigeria reported 87% awareness level but only 27% vaccination coverage [22], while, in north central Nigeria, Okeke et al. reported that only 48% completed their HBV vaccination with an awareness level of 92% [23]. The reason between the level of awareness and vaccination in the study by Okeke et al. was due to lack of opportunity and forgetting to be vaccinated, while Okwara reported high response among those that had tertiary education. About 99% of our respondents had tertiary education with a vaccination rate of 65%. This supports the findings of Okwara et al. [13], even though we did not inquire into reasons why they did not take vaccine. In addition, we also found that being a male and having had a previous HBsAg test were strongly associated with HBV vaccination.

The situation is however different outside Nigeria with higher vaccine coverage among health workers [24, 25]. This may therefore translate to routine HBsAg and anti-HBs tests and vaccination for Nigerian health workers who wish to practice in other countries of the world.

Only 37% were screened for HBsAg before vaccination. The government of Nigeria as well as the institution's policy is to vaccinate everyone without a prior HBsAg test. However, the liver unit of the hospital advise that screening is done before vaccination so that only those who will benefit take the vaccine. The practice of vaccination without HBsAg and anti-HBs tests can give a false vaccine protection to infected people thereby making them prone to the chronic complications of the virus. Therefore, a mandatory Nigerian government and hospital policy of HBsAg screening and vaccination may need to be put in place for workers and patients protection.

The number that had treatment or those that visited a doctor after a positive HBsAg test was small. This again may reflect their poor knowledge of HBV infection and its long term complications as has been shown in this study.

In this study, factors that favoured a good HBV knowledge were slightly different from that reported by Karaivazoglou et al. among health care workers in Greece [25], while they reported occupation, higher education, and HBsAg vaccination; this study in addition identified younger age, male sex, and having had a previous HBsAg test as indices of good knowledge of HBV.

Several studies have highlighted the importance of the control of viral hepatitis through health education, hepatitis B vaccination of at risk population, and treatment of infected persons [7, 9, 26–28]. Thus, the lack of adequate hospital policy to enforce mandatory hepatitis B test as well as its poor implementation may hinder the effective control of HBV. Many workers that started HBV vaccine did not complete probably due to fear or side effects of the vaccine.

The reported positive association between knowledge of HBV and vaccine uptake as well as poor orientation of new members of staff among the respondents may result from low risk perception which increases possibility of HBV infection through obscure routes. It therefore implied that all health care workers should be educated on HBV and the necessity of following the HBV vaccine regimen.

Limitation to this study is that it is a cross-sectional study; therefore, cause-effect relationship may be difficult to establish. Some respondents could also have given socially acceptable responses to some questions even though they were reassured about the purpose of the study.

## 5. Conclusion

The hospital workers of this institution have low perceived risk of HBV infection and low vaccination coverage despite a high awareness of HBV vaccine. Therefore, a policy of mandatory HBsAg screening and vaccination may need to be put in place to protect both staff and patients of the institution. Free HBsAg screening for newly employed staff before vaccination may need to be incorporated into the policy to make it effective.

## Figures and Tables

**Table 1:** Sociodemographic characteristics of respondents.

Characteristics	Sex		Total (%)
	Male (%)	Female (%)	
Age group (years)			
18–34	110 (49)	114 (51)	224 (59)
35 and above	72 (46)	86 (54)	158 (41)
Marital status			
Single	97 (58)	69 (42)	166 (43)
Married	85 (39)	131 (61)	216 (57)
Highest level of education			
Secondary	3 (100)	0 (0)	3 (0.8)
Tertiary	179 (47)	200 (53)	379 (99)
Occupation			
Medical doctor	73 (78)	21 (22)	94 (25)
Nurse	40 (24)	128 (76)	168 (44)
Laboratory technologist	43 (63)	25 (37)	68 (18)
Pharmacist	26 (50)	26 (50)	52 (14)
Religion			
Christianity	143 (45)	175 (55)	318 (83)
Islam	39 (61)	25 (39)	64 (17)
Ethnicity			
Yoruba	156 (46)	184 (54)	340 (89)
Igbo	15 (63)	9 (38)	24 (6)
Hausa/Urhobo	11 (61)	7 (39)	18 (5)
HBsAg result			
Positive	9 (4.9)	9 (4.5)	18 (4.7)
Negative	119 (65.4)	130 (65)	249 (65.2)
Not done	54 (29.7)	61 (30.5)	115 (30.1)

**Table 2:** Knowledge, routes, and means of infection and at risk population of HBV.

Variables	Medical doctors (%)	Nurses (%)	Lab technologists (%)	Pharmacists (%)	*P* value
Aware of HBV (yes)	93/94 (99)	159/168 (95)	64/68 (94)	51/52 (98)	0.829
HBV causes liver cancer? (Yes)	92/94 (98)	107/168 (64)	44/68 (65)	30/52 (58)	0.001
HBV causes liver cirrhosis (yes)	91/94 (97)	132/168 (79)	56/68 (82)	40/52 (77)	0.001
Infection routes					
Blood (yes)	92/94 (98)	163/168 (97)	64/68 (94)	51/52 (98)	0.587
Tear (yes)	37/94 (39)	41/168 (24)	16/68 (24)	13/52 (25)	0.046
Saliva (yes)	55/94 (59)	75/168 (45)	28/68 (41)	23/52 (44)	0.127
Vaginal/seminal fluid (yes)	25/94 (27)	126/168 (75)	49/68 (72)	43/52 (83)	0.001
Means of HBV infection					
Exchange of needle (yes)	94/94 (100)	143/168 (85)	56/68 (82)	50/52 (96)	0.001
Vertical transmission (yes)	86/94 (92)	129/168 (77)	51/68 (75)	34/52 (65)	0.001
At risk population					
^*^MSM (yes)	91/94 (97)	111/168 (66)	44/68 (65)	38/52 (73)	0.001
Sex workers (yes)	93/94 (99)	133/168 (79)	49/68 (72)	46/52 (89)	0.001
Health workers (yes)	94/94 (100)	154/168 (92)	59/68 (87)	49/52 (94)	0.001
Long distance drivers (yes)	89/94 (95)	63/168 (38)	18/68 (27)	13/52 (25)	0.001
Injection drug users (yes)	94/94 (100)	140/168 (83)	43/68 (63)	41/52 (79)	0.001
Sickle cell anaemic patients (yes)	70/94 (75)	71/168 (42)	19/68 (28)	15/52 (29)	0.001
Multiple sexual partners (yes)	91/94 (97)	130/168 (77)	47/68 (69)	44/52 (85)	0.001

^*^MSM: men having sex with men.

**Table 3:** HBV serostatus, awareness, and vaccination history of respondents.

Variable	Medical doctors (%)	Nurses (%)	Lab technologists (%)	Pharmacists (%)	*P* value
Ever screened for HBV (yes)	76/94 (81)	118/168 (70)	55/68 (81)	18/52 (35)	0.001
Knows present status	66/76 (87)	82/118 (70)	46/55 (84)	14/18 (78)	0.024
Took action on positive HBV test (yes)	1/2 (50)	7/12 (58)	3/5 (60)	1/1 (100)	1.000
Completed HBV vaccination after negative HBV test	59/64 (92)	61/70 (87)	27/41 (66)	13/13 (100)	0.002
Proportion on treatment	1/2 (50)	7/12 (58)	2/3 (67)	1/1 (100)	1.000
Proportion that completed treatment	1/1 (100)	4/7 (57)	2/3 (67)	1/1 (100)	1.000
Proportion that cleared HBV	1/1 (100)	1/4 (25)	2/3 (67)	1/1 (100)	0.571
Aware of HBV vaccine (yes)	93/94 (99)	164/168 (98)	65/68 (96)	50/52 (96)	0.474
Received 3 doses of HBV vaccine	80/94 (85)	87/168 (52)	46/68 (68)	35/52 (67)	0.001
Screened for HBsAg before vaccination	43/86 (50)	54/127 (43)	38/56 (68)	8/45 (18)	0.001
Willingness to receive HBV vaccination	82/84 (98)	144/155 (93)	61/63 (97)	48/49 (98)	0.349

**Table 4:** Multivariate analysis of factors associated with good knowledge of HBV among the respondents.

Variable	AOR	95% CI	*P* value
Age group (years)			
18–34	1.746	1.130–2.700	0.012
≥35	1		
Sex			
Male	1.984	1.172–3.359	0.011
Female	1		
Marital status			
Married	0.747	0.485–1.151	0.187
Single	1		
Occupation			
Medical doctor	24.057	6.731–85.978	0.001
Nurse	1.110	0.593–2.079	0.744
Lab technologist	0.841	0.407–1.737	0.640
Pharmacist	1		
Ever screened for HBsAg			
Yes	2.021	1.205–3.389	0.008
No	1		
Doses of vaccine taken			
Appropriate	2.000	1.290–3.103	0.002
Inappropriate	1		

**Table 5:** Multivariate analysis of factors associated with HBV vaccine uptake among health workers.

Variable	AOR	95% CI	*P* value
Age group (years)			
18–34	1.681	0.947–2.986	0.076
≥35	1		
Sex			
Male	1.756	1.067–2.890	0.027
Female	1		
Marital status			
Married	1.718	0.917–3.218	0.091
Single	1		
Occupation			
Medical doctor	1.037	0.172–6.242	0.968
Nurse	0.501	0.212–1.184	0.115
Lab technologist	0.420	0.158–1.114	0.081
Pharmacist	1		
Ever screened for HBsAg			
Yes	3.689	2.078–6.547	0.001
No	1		

**Table 6:** Challenges to control of hepatitis B infection among health workers.

^*^Challenges	Frequency (*N* = 382)	%
Inappropriate hospital policy	350	91.6
Poor orientation of new health workers	290	75.9
Low risk perception	285	74.6
Poor knowledge	124	32.5
Poor implementation of hospital policy	112	29.3
Fear of side effects of vaccine/injection	85	22.3

^*^Multiple response.
